# Unjust: the health records of youth with personal/family justice involvement in a large pediatric health system

**DOI:** 10.1186/s40352-021-00147-5

**Published:** 2021-08-01

**Authors:** Samantha Boch, Emre Sezgin, Donna Ruch, Kelly Kelleher, Deena Chisolm, Simon Lin

**Affiliations:** 1 College of Nursing, University of Cincinnati, Cincinnati, OH USA; 2 James M. Anderson Center for Health Systems Excellence, Cincinnati Children’s Hospital Medical Center, Cincinnati, OH USA; 3 Abigail Wexner Research Institute at Nationwide Children’s Hospital, Columbus, OH USA; 4 Department of Pediatrics, The Ohio State University College of Medicine, Columbus, OH USA

**Keywords:** Child health, Incarceration, Medical records, Justice-involvement

## Abstract

**Background:**

Mass incarceration has had an undeniable toll on childhood poverty and inequality, however, little is known about the consequences on pediatric health. The purpose of this study was to identify and describe the health of pediatric patients with probable personal or family history involvement with the correctional system.

**Methods:**

A descriptive study was conducted using electronic health record data of 2.3 million youth (ages 0–21 years) who received care in a large Midwestern hospital-based institution from February 2006–2020. We employed a correctional-related keyword search (e.g. jail, prison, probation, parole) to locate youth with probable personal or family history involvement. Health characteristics were measured as clinician diagnostic codes.

**Results:**

Two percent of the total pediatric population had a correctional keyword in the medical chart (*N* = 51,855). This 2% made up 66% of all patients with cannabis-related diagnoses, 52% of all patients with trauma-related diagnoses, 48% of all stress-related diagnoses, 38% of all patients with psychotic disorder diagnoses, and 33% of all suicidal-related disorders within this institution’s electronic health record database – among other highly concerning findings.

**Conclusions:**

We captured an alarming health profile that warrants further investigation and validation methods to better address the gaps in our clinical understanding of youth with personal or family history involvement with the correctional system. We can do better in identifying, and supporting families affected by the correctional system.

## Introduction

The size and churn of the correctional system in the United States is staggering. In 2016, alone, over 815,000 youth had contact with the juvenile justice system (Sickmund et al., 2020), and nearly 6.6 million adults were on probation or parole, or in jail or prison (Kaeble & Cowhig, 2018). While incarceration rates have slowly declined over the past decade (Kaeble & Cowhig, 2018), about 600,000 people are sentenced to prison, and 4.9 million are detained in jails *every year* in the U.S*.* (Sawyer & Wagner, 2020). Over time, this has resulted in 77 million criminal records (Sawyer & Wagner, 2020) and numerous collateral consequences for family and community health. The traumatic separation of a child from their parent via incarceration can ensue multiple household, custodial/caregiver, and economic disruptions upon arrest and throughout incarceration. Upon release of incarceration, a record of crime can restrict where a family can live and work, and in some states, can even restrict their eligibility for government aid (Turney & Goodsell, 2018). Our correctional system has undeniably shaped the landscape of child inequality (Bowleg, 2020; Wildeman et al., 2017), and has disproportionately affected families of color, families in poverty, and families in rural areas for centuries (Blankenship et al., 2018; Murphey & Cooper, 2015). Yet, we know very little about the clinical health records of youth with personal or family justice-involvement because of inadequate cross sector collaborations and investigations.

While many youths have varying levels of personal or family contact with the justice system in the US, we know most about the health and well-being of incarcerated youth. Recent systematic reviews and meta-analyses on the health of incarcerated or previously incarcerated youth have revealed higher prevalence rates of self-harm, risky behavior, neurodevelopmental disabilities, infectious disease, adolescent morbidity, adolescent mortality, and psychiatric disorders (with anxiety, mood, and substance use disorders most common) compared to youth with no contact to the justice system (Beaudry et al., 2021; Borschmann et al., 2020; Livanou et al., 2019). Youth who leave juvenile detention centers experience lingering psychiatric conditions (Teplin et al., 2021), poor physical health and functioning (Barnert et al., 2017), in addition to legal, socioeconomic, and educational challenges across the lifespan (Farrington et al., 2018). Researchers in the field have called for more high-quality data and rigor in research (Borschmann et al., 2020), and more information on female youth (Beaudry et al., 2021) and youth of various ethnicities (Livanou et al., 2019).

A smaller body of research exists on the health of youth who have been exposed to a parent’s incarceration. Research has documented linkages between youth ever exposed to parental incarceration and child mortality (Wildeman, 2012), elevated risks of drug use and abuse (Roettger et al., 2011), delinquency (Porter & King, 2015), poor health status, learning disabilities, developmental delays, and various mental health problems (e.g. externalizing, internalizing, and attention difficulties) and conditions (e.g. attention deficit disorder, depression, anxiety, conduct problems) compared to youth unexposed (Boch et al., 2019; Boch & Ford, 2018; Turney, 2014; Wildeman et al., 2018). Similar to the gaps in literature on incarcerated youth, researchers have argued the need for greater interdisciplinary investigation and higher quality data using administrative health records (Wildeman et al., 2017; Wildeman et al., 2018).

Even fewer studies exist on the health of youth with other types of family contact with the system. Of those that do exist, most focus on the association of sibling incarceration and their linkages to personal criminal involvement (Wagner et al., 2014) or poor school outcomes (Nichols & Loper, 2012). Because the US locks up the greatest proportion of the world’s incarcerated (Walmsley, 2018), much greater attention and structural investment is needed to understand the health of youth with varying exposures to the justice system. The consequences of mass incarceration in the US have limited opportunities for many children, families, and communities (Wildeman, 2009; Wildeman & Wang, 2017) – described as hidden consequences (Martin, 2017), a public health crisis (Cloud et al., 2014), and a threat to health equity (Acker et al., 2019; Bowleg, 2020). Due to the advent of large information databases and systems, locating youth with varying levels of exposure to the justice system using existing electronic health records of large pediatric health care systems can now be explored. To take one step towards uncovering the clinical health of these youth, an explorative descriptive study was conducted using the electronic health record database of a large Midwestern pediatric hospital-based institution.

## Methods

### Setting

We queried EPIC medical records on 2.3 million youth (under 21 years of age) in the electronic health record database of a large Midwestern pediatric hospital-based institution from February 2006 to February 2020, using natural language processing. The institution provides care for more than 1.5 million patient visits annually from all 50 states and over 45 countries. The hospital-based system includes a network of primary care centers, behavioral health clinics, urgent care clinics, two emergency departments and 527 inpatient beds on main campus, plus 146 offsite inpatient beds as part of its neonatal network.

### Data query details

For our query, we used a natural-language processing supported search engine to extract similar keywords (Moosavinasab et al., 2021) related to prison, jail, probation, and parole to include all correctional-related keywords used by clinicians in the notes. We chose these terms to capture all four types of correctional detainment following arrest in the United States. Similar keywords were then pulled (by natural language processing) from this database of clinician notes and yielded the terms: “sentenced”, “imprisoned”, and “incarcerated.” We also included “parent” keywords to capture the health records of youth exposed to parental incarceration. The finalized data query included the following keywords: [(“parent” OR “mom” OR “mother” OR “dad” OR “father”) AND (“incarcerated” OR “imprisoned” OR “sentenced” OR “jail” OR “prison” OR “parole” OR “probation”). Any type of clinician note in the medical record were eligible to be searched. The lone exception was a tuberculosis risk assessment text field where “incarcerated adolescents” auto-populated as a reminder query for high-risk contact to decrease the chances of false-positives.

### Diagnostic measures

All patient health characteristics were measured as clinician diagnostic codes via the International Classification of Diseases Version 9/Version 10 (ICD) and current procedural terminology (CPT) codes. Data captured indicates any related-diagnosis within the 14-year time span and counted each as a single occurrence under the condition (e.g. generalized “anxiety” disorder, unspecified, and/or generalized “anxiety” disorder, severe, and/or phobic “anxiety” disorder, and/or other “anxiety” disorder). Relevant disorder keywords were selected based on the following criteria: 1) highest prevalence associated with the selected correctional-related keywords yielding: *acute respiratory infection, allergic rhinitis, anemia, mild intermittent asthma, dermatitis, obesity, anxiety, attention deficit, conduct disorder, depressive disorders, suicide, otitis media* and the following CPT/ICD codes: *level 4 office visit, level 5 emergency department visit, child in welfare custody, caregiver refusal of immunization, unspecified lack of expected physiological development, encounter for routine child health examination with abnormal findings, screening for sexually transmitted infections, and exposure to environmental tobacco smoke;* or, 2) known to be associated with *toxic stress* (e.g. *trauma, post-traumatic stress, adjustment disorder, elevated blood pressure reading, overweight, failure to thrive*), or 3) known to be associated with parental incarceration (e.g. *obesity, developmental disorders, behavioral conditions such as depression, anxiety, conduct*), or 4) known to be associated with juvenile incarceration (e.g. *trauma, cannabis/nicotine/alcohol use, bipolar, psychosis, adjustment disorder, substance use disorder*). *Toxic stress* is defined as the overwhelming, frequent, or prolonged stress response without sufficient buffering of a stable responsive adult (Shonkoff et al., 2012) which many children of incarcerated parents may encounter if their only primary caregiver, or both of their parents are incarcerated.

### Statistical analysis

Summary statistics were used to describe the demographic and diagnostic characteristics of youth identified by the correctional keyword supported search and the total population. We also calculated a trend line to describe the number of new patients with a documented correctional keyword in their electronic health record over the number of total patients at the institution across time. In addition, we extracted 1000 *random* clinician notes for two coauthors to review and annotate for type of personal or family correctional involvement (to provide a snapshot of the various exposures to the system documented at this institution). This sample size derived from a population of approximately 52,000 notes/unique youth allows the estimation of a 95% confidence interval with a +/− 3% margin of error. All study procedures were approved by the hospital’s Institutional Review Board.

## Results

### Patient characteristics

**Fig. 1:**
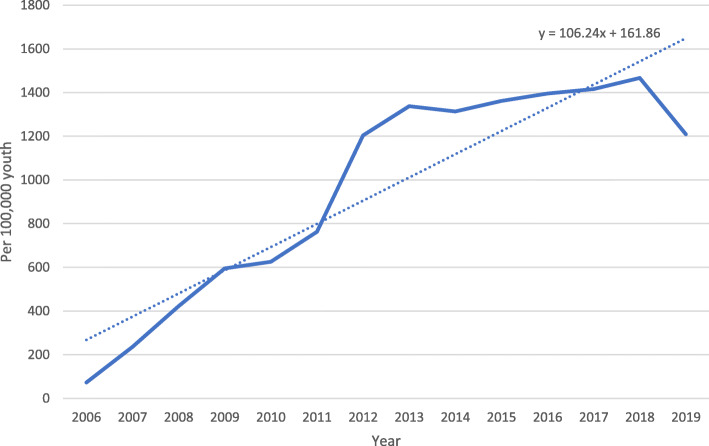
Number of new patients with a documented correctional keyword in their electronic health record over the number of total patients seen (per 100,000) by year from 2006 to 2019. Source: Authors’ analysis of data. Y-axis represents the number of unique patients with a newly documented correctional keyword in their electronic health record out of total patients treated (each year) at a large Midwestern pediatric hospital-based institution across time (February 2006–December 2019). Total population of database = 2.3 million unique patients. Note: Criminal justice related query words used in keyword supported search: (“incarcerated” OR “imprisoned” OR “jail” OR “prison” OR “sentenced” OR “parole” OR “probation”)

Figure 1 depicts the number of new patients with a documented correctional keyword (e.g. prison, jail, parole, or probation) out of the total number of patients treated (per 100,000) by year. As depicted, we found a steady increase in the incidence rate of pediatric patients with a provider documented correctional keyword in their chart across time.

Table 1 summarizes the demographics of patients identified by the keyword search, and total patients in the database. About 2% of patients (51,855 patients out of 2.3 million) had a correctional keyword within their records. Nearly half (*n* = 27,167 or 52.4%) of youth with a correctional keyword identified as male, and nearly half (*n* = 26,576 or 51.3%) were white. Age was calculated at the time of the data pull and therefore, most of the patients identified by the keyword search were ages 13 and older (51.4%). Most youth (87.7%) had Medicaid/SCHIP as their health insurance coverage (*n* = 45,479).

**Table 1:** Demographic and health utilization of patients identified by correctional/family keywords and all patients (ages 0–21) in electronic health record (EHR) database from February 2006–2020

Patient Characteristics	Patients with Correctional Keywords in the EHR ***n*** = 51,855		All Patients in Database ***N*** = 2,337,632		% of All Patients with Correctional Keyword* (n^**a**^/n^**b**^)
	n^**a**^	%	n^**b**^	%	%
**Gender**					
Female	24,674	47.58%	1,155,026	49.40%	2.14%
Male	27,167	52.39%	1,182,606	50.60%	2.30%
Age Range**					
0–4 years	6537	12.61%	224,158	9.60%	2.92%
5–9 years	11,722	22.61%	288,067	12.30%	4.07%
10–12 years	6913	13.33%	178,947	7.70%	3.86%
13–18 years	13,911	26.83%	328,909	14.10%	4.23%
19+ Years	12,764	24.61%	1,318,912	56.40%	0.97%
**Race**					
Black/African American	16,905	32.60%	309,130	13.20%	5.47%
White	26,576	51.25%	1,204,313	51.50%	2.21%
Unknown	2159	4.16%	687,911	29.40%	0.31%
Multiple Race	4596	8.86%	53,089	2.30%	8.66%
Asian Race	637	1.23%	36,514	1.60%	1.74%
Other	141	0.27%	18,699	0.80%	0.75%
Native Hawaiian	266	0.51%	13,756	0.60%	1.93%
Refuse to Answer	2159	4.16%	9881	0.40%	21.85%
American Indian or Alaskan Native	74	0.14%	5694	0.20%	1.30%
**Ethnicity**					
Not Hispanic or Latino	47,978	92.52%	1,607,625	68.80%	2.98%
Hispanic or Latino	2353	4.54%	44,912	1.90%	5.24%
Health Insurance Coverage***					
Medicaid/SCHIP	45,479	87.70%	618,899	26.50%	7.35%
Private/Commercial	13,986	26.97%	635,021	27.20%	2.20%
Self-Pay	15,294	29.49%	176,528	7.60%	8.66%
Other-Unknown	4896	9.44%	21,006	0.90%	23.31%
Medicare	179	0.35%	5812	0.20%	3.08%

Notes: Authors’ analysis of electronic health record data of a large Midwestern pediatric hospital-based institution. (N = 2.3 million unique patients ages 0–21 years). Correctional query words algorithm used in keyword supported search: (“incarcerated” OR “imprisoned” OR “jail” OR “prison” OR “sentenced” OR “parole” OR “probation”). *Number of patients with the given characteristic and a correctional keyword in their medical chart out of the total population. **Age range indicates current age of the youth in the system and not age at time of possible exposure to family history or personal history of correctional involvement. ***Health insurance coverage: counts do not add up as there can be multiple types of insurance used for visit/per patient

### Patient diagnoses

Table 2 summarizes the diagnoses of the patients identified by the keyword search and total patients in the database. The top five physical health related diagnoses in youth with a correctional keyword (*n* = 51,855) included acute upper respiratory infection-related disorders (40.0%, *n* = 20,740), otitis media-related disorders (37.1%, *n* = 19,232), contact dermatitis related disorders (31.9%, *n* = 16,559), asthma-related disorders (24.7%, *n* = 12,781) and allergic-rhinitis related disorders (22.3% or *n* = 11,572). Even though youth with a correctional keyword constituted roughly 2% of the total number of patients (*N* = 2.3 million), they made up a moderate proportion of all youth diagnosed with a physical health disorder including 35.5% of all anemia-related disorders (6636 out of 18,682), 17.0% of all developmental related disorders of speech and language (261 out of 1537), 16.7% of all elevated blood pressure-related codes (3625 out of 21,724), 14.0% of all overweight related codes (3995 out of 28,613), and 13.3% of all allergic-rhinitis related disorders (11,572 out of 86,781).

**Table 2:** Health and health care characteristics of patients identified by correctional/family keywords and all patients (ages 0–21) in electronic health record (EHR) database from February 2006–2020

Health Diagnoses, Health Characteristics, and Health Care Use	Patients with Correctional Keywords in the EHR n = 51,855		All Patients in Database N = 2,337,632		% of All Patients with Correctional Keyword* (n^**a**^/n^**b**^)
	n^**a**^	%	n^**b**^	%	%
**Physical Health**					
*Acute upper respiratory infection*-related	20,740	40.00%	194,575	8.30%	10.66%
*Allergic rhinitis*-related	11,572	22.32%	86,781	3.70%	13.33%
*Anemia*-related	6636	12.80%	18,682	0.80%	35.52%
*Mild intermittent asthma*-related	12,781	24.65%	120,353	5.10%	10.62%
*Cardiac murmur*-related	2533	4.88%	28,589	1.20%	8.86%
*Congenital heart disease*-related	222	0.43%	2732	0.12%	8.13%
*Dermatitis*-related	16,559	31.93%	130,379	5.60%	12.70%
*Developmental disorders of speech* and language - related	261	0.50%	1537	< 0.001	16.98%
*Elevated blood-pressure reading* –related	3625	6.99%	21,724	0.92%	16.69%
*Failure to thrive* - related	3456	6.66%	27,336	1.20%	12.64%
*Obesity*- related	8514	16.42%	65,976	2.80%	12.90%
*Otitis media*-related	19,232	37.09%	215,198	9.20%	8.94%
*Overweight*- related	3995	7.70%	28,613	1.20%	13.96%
**Psychiatric Health**					
*Alcohol disorder*-related	191	0.37%	445	0.02%	42.92%
*Adjustment disorder*-related disorders	4567	8.81%	15,916	0.68%	28.69%
*Anxiety* related disorders	11,799	22.75%	64,224	2.70%	18.37%
*Attention-deficit & hyper* related disorders	13,151	25.36%	57,886	2.50%	22.72%
*Bipolar* -related	1884	3.63%	6921	0.30%	27.22%
*Cannabis disorder* -related	672	1.30%	1015	0.04%	66.21%
*Conduct disorder*- related	5952	11.48%	18,254	0.80%	32.61%
*Major depressive*- related	7194	13.87%	25,048	1.10%	28.72%
*Nicotine disorder* -related	79	0.15%	167	0.01%	47.31%
*Psychosis* related	836	1.61%	2222	0.10%	37.62%
*Post-traumatic stress*-related	4121	7.95%	8618	0.37%	47.82%
*Substance use disorder* - related	173	0.33%	321	0.01%	53.89%
*Suicide or suicidal* - related	7021	13.54%	21,408	0.92%	32.80%
*Trauma-related disorder* -related	1260	2.43%	2431	0.10%	51.83%
**Social and Environmental**					
Exposure to environmental tobacco smoke (Z77.22)	4291	8.27%	21,042	0.90%	20.39%
Screening for STIs (Z11.3)	2284	4.40%	18,309	0.80%	12.47%
Encounter for routine child health examination with abnormal findings (Z00.121)	4944	9.53%	32,805	1.40%	15.07%
Unspecified lack of expected physiological development (R62.50)	2895	5.58%	21,739	0.90%	13.32%
Child in welfare custody (Z62.21)	1836	3.54%	4424	0.20%	41.50%
Caregiver refusal of immunization (Z28.82)	1329	2.56%	8144	0.30%	16.32%
Health Care Use**					
Level 4 Office Visit (CPT 99214)- *Office or other outpatient visit for the evaluation and management of an established patient, which requires at least 2 of the 3 CPT qualifying components.*	30,139	58.14%	292,761	12.5%	10.29%
Level 5 Emergency Department Visit (CPT 99285): *Emergency department visit for the evaluation and management of a patient, which requires these 3 key components within the constraints imposed by the urgency of the patient’s clinical condition and/or mental status.*	20,825	40.17%	161,713	6.90%	12.88%

Notes: Authors’ analysis of electronic health record data of a large Midwestern pediatric hospital-based institution. (N = 2.3 million unique patients ages 0–21 years). Correctional query words algorithm used in keyword supported search: (“incarcerated” OR “imprisoned” OR “jail” OR “prison” OR “sentenced” OR “parole” OR “probation”). All patient health characteristics are represented International Classification of Diseases Version 9 or Version 10 codes and the italicized words indicate the diagnostic keyword searched within the medical record unless the specific ICD/CPT code is listed. Diagnostic codes and characteristics are not mutually exclusive. *Number of patients with the given health characteristic and correctional keyword out of the total population. **Healthcare utilization was determined by current procedural terminology (CPT) codes

The top five psychiatric disorders in youth with a correctional keyword (*n* = 51,855) were attention deficit-related disorders (25.4%, *n* = 13,151), anxiety-related disorders (22.8%, *n* = 11,799), depression-related disorders (13.9%, *n* = 7194), suicide-related disorders (13.5%*, n* = 7021), and adjustment-related disorders (8.8%, *n* = 4567). Even though youth with a correctional keyword constituted roughly 2% of total number of patients, they made up a large proportion of all patients with certain diagnosed psychiatric disorders including 66.2% of all patients with cannabis-related disorders (672 out of 1015), 53.9% of all patients with substance use-related disorders (173 out of 321), 51.8% of all patients with trauma-related disorders (1260 out of 2431), 47.8% of all patients with stress-related disorders (4121 out of 8618), and 37.6% of all patients with psychotic-related disorders (836 out of 2222).

Unsurprisingly, these youth made up nearly half of all children in welfare custody (41.5% or 1836 out of 4424 youth). However, in regards to health care use, youth who had a documented correctional keyword also made up a sizeable proportion of all time-intensive office visits and emergency department visits, 10.3% of all level 4 office visits (CPT code 99214; 30,139 out of 292,761) and 12.9% of all level-5 emergency department visits (CPT code: 99285; 20,825 out of 161,713).

### Chart validation of query: types of correctional involvement

About 83% of the 1000 clinician notes that were examined for validation indicated some type of personal or family involvement with the correctional system. Of the 1000 notes, 310 indicated father figure involvement, 160 indicated mother figure involvement, 69 indicated “other family” (e.g. sibling, cousin, grandparent), 287 indicated youth/self, 34 indicated partner involvement (of the parent or youth), 45 indicated generational or multiple types, and 173 indicated no involvement (e.g. child described feelings of “being in prison” or medical condition such as “incarcerated” hernia).

## Discussion

Our results depict a highly concerning diagnostic profile in a small proportion of youth treated at this institution with probable exposure to the correctional system. Only 2% of all youth treated at this institution had a clinician-documented correctional keyword in their chart, yet, they accounted for nearly 1 in every 3 patients with selected/related psychiatric disorders and nearly 1 in every 10 patients with selected/related physical health disorders*.* To our knowledge, this is the first study describing the aggregation of health record data on youth with probable personal or family exposure to the justice system (using existing clinician notes). Because the majority of those incarcerated in our correctional system are parents (Glaze & Maruschak, 2008), and because the majority of those who are incarcerated (as youth or adults) are more likely to have previous trauma and abuse, addiction, and be reared in poverty (Binswanger & Elmore, 2015) – our study findings suggest that children and families of those who are incarcerated could use additional follow-up. Youth are especially vulnerable to chronic stressors and strain due to sensitive neurodevelopment architecture (Shonkoff et al., 2012). Other contributing factors that may have led to the poor health in these youth may relate to the social risk factors that led to their, or their parent’s incarceration (e.g. poverty, drug use and addiction, trauma, neighborhood violence), the trauma associated with incarceration, the neglect/abuse/victimization related to a parent’s incarceration, the displacement of a child to foster care or to new caregiver, and the societal stigma, shame, and legal discrimination post-incarceration. Future research must refine and replicate these methods to adjust for social adversity to better understand and compare findings with matched-comparator groups of youth living among similar social and community risk factors, and to inform policy. Leveraging other big data methods such as machine learning to locate children of by type of exposure (e.g. children of incarcerated parents) for cohort identification and observational research could also fulfill identified gaps in the literature. Using such identification approaches could also be used to link families to helpful community resources and referrals, and to guide supportive follow-up in addition to clinician and family decision-making.

Taken together, our study underscores the urgency of identification of youth exposed to family or personal correctional involvement in the electronic health record and greater investigation on the ways to screen and provide better care for these children. While parental or self-incarceration is included in some adverse childhood experiences checklists as survey items, few pediatric health providers and systems routinely screen for adverse childhood experiences (Kerker et al., 2016) or other social determinants of health (Fraze et al., 2019) that may not only be helpful in the prevention of poor health, but the prevention of justice involvement. Because we do not consistently or routinely screen for types of exposure to the correctional system in pediatric health care systems, we know little about these youth and their families using medical record data and meaningful ways to intervene. However, as we await wide-scale implementation of such screenings, leveraging existing medical data can help fulfill the gaps in our sciences on these youths.

Other mounting evidence also confirms we have great need for timely identification. Two recent systematic reviews confirm the associations between parental incarceration and poor child health (Boch & Ford, 2018; Wildeman et al., 2018), in addition to the literature that details the poor mental health of justice-involved youth (Balogun et al., 2018; Owen & Wallace, 2020; Underwood & Washington, 2016). However, few of these studies investigated the causal mechanisms or effective interventions across developmental stages. The toxic stress (Garner et al., 2012; Shonkoff et al., 2012) associated with any involvement of the correctional system, whether from personal or family contact, is likely a mechanism worthy of further study in our youth. If we are truly protecting the nation with our correctional system, we must ensure that the families tangentially affected are also protected and supported. As recommended by the National Academies of Science, Engineering, and Medicine (2019), *greater collaboration among our health, justice, and child welfare systems* is needed in order to provide opportunities for all youth to thrive and flourish. As our findings suggest, there are compelling reasons for doing so. Greater cross-sector collaboration requires the perspective and engagement of families affected. However, centuries of structural racism embedded in our health and justice systems may actually prevent families from participating in such efforts due to historical distrust. In addition, legal barriers may even preclude youth and families with histories of incarceration from participating as engaged family partners - which will be critical to address if we continue to research families and children affected by any history of incarceration.

Finally, it is important to note that the use and application of big data methods to address the needs of our justice system are widely investigated, theorized, and contested across disciplines and advocacy groups (Završnik, 2019). However, the use of keyword searches and other natural language processing approaches within electronic health records are novel (Hanauer et al., 2015) and definitely warrants similar investigation and ethical scrutiny. Most institutional review boards have regulatory procedures and special review processes to ensure that justice-involved youth and adults who participate in research are doubly protected because of their vulnerabilities, but youth who have family members who are justice involved are not typically considered.

### Limitations

First, this is a population seen at one pediatric hospital institution in a Midwestern metropolis and includes only their electronic health record data. As a result, generalizability may be limited to similar settings and similar patient populations. In addition, any diagnoses outside of the hospital system may not have been captured. This is particularly relevant for children and families who are justice involved due to their increased likelihood to move due to caregiver or other custodial changes. However, our sample size and time frame provide a novel contribution and call to action. Second, we searched for all related disorder codes and counted each as a single occurrence under the condition (e.g. generalized “anxiety” disorder, unspecified, and/or generalized “anxiety” disorder, severe) which could lead to over-counting especially if a provider listed a rule-out diagnosis type associated with the condition. Third, counts of correctional involvement is likely underestimated in our population as patients and families are not routinely screened for involvement with the correctional system. In addition to only capturing the providers who felt that it was important to document, families often refrain from disclosing such information out of fear of judgement and stigma. In combination, these results only portray a proxy of unverified and potential exposure to family or personal correctional involvement and a proxy of related health disorder fields. Replication of this work using other health record databases, refined correctional keywords (e.g. the inclusion of “arrest” or other justice based words that are geographic-based), refined ICD groupings, and inclusion of matched comparator groups (to capture social, neighborhood and other upstream factors that relate to both incarceration and poor health) are warranted to assist with causal inferences and sense-making. In addition, we acknowledge the reciprocal nature of mass incarceration and community deprivation which make understanding these relationships methodologically challenging. Despite these limitations, we feel strongly that this descriptive study is highly innovative and that the “mark” of any contact with the justice system is certainly worthy to investigate considering the size and churn of our correctional system and its embedded structural racism.

## Conclusion

We can do better in identifying, and supporting families tangentially affected by the correctional system. Determining whether mass incarceration has more negative net public health effects on children is an important public health and justice issue that can no longer be ignored. More importantly, if the results are confirmed, the urgency of preventive interventions for children affected by the correctional system cannot be overstated, especially for their behavioral health.

## Data Availability

The dataset generated and analyzed during the current study are not publicly available as these are pediatric medical records of a large health system and protected under the Health Insurance Portability and Accountability Act.
